# Graded motor imagery for women at risk for developing type I CRPS following closed treatment of distal radius fractures: a randomized comparative effectiveness trial protocol

**DOI:** 10.1186/s12891-018-2115-6

**Published:** 2018-06-26

**Authors:** Corey McGee, Jennifer Skye, Ann Van Heest

**Affiliations:** 1 Programs in Occupational Therapy and Rehabilitation Science, Center for Allied Health Programs, Medical School, University of Minnesota, MMC 368, 420 Delaware St. SE, Minneapolis, MN 55455 USA; 2 Program in Rehabilitation Science, Medical School, University of Minnesota, Minneapolis, MN USA; 3 Department of Orthopaedic Surgery, Medical School, University of Minnesota, Minneapolis, MN USA

**Keywords:** Distal radius fracture, Graded motor imagery, Mirror therapy, Movement representation techniques, Complex regional pain syndrome, Non-operative, Immobilization, Cast, Women, Clinical trial

## Abstract

**Background:**

Distal radius fractures (DRF) account for nearly one-fifth of all fractures in older adults, and women experience them 5× as often as men. Most DRF occur with low impact injuries to the wrist with an outstretched hand, and are often managed via closed treatment and cast immobilization. Women sustaining a DRF are at risk for upper limb immobility, sensorimotor changes, edema and type I complex regional pain syndrome (CRPS). Since CRPS onset is likely influenced by alterations in the brain’s somatosensory region, a rehabilitation intervention, Graded Motor Imagery (GMI), aims to restore cortical representation, including sensory and motor function, of the affected limb. To date, there are no studies on the use of GMI in reducing risk of or preventing the onset of type I CRPS in women with DRF treated with cast immobilization. Due to a higher likelihood of women with this injury developing type I CRPS, it is important to early intervention is needed.

**Methods/design:**

This article describes a six-week randomized comparative effectiveness trial, where the outcomes of a modified GMI program (mGMI) + standard of care (SOC) group (*n* = 33) are compared to a SOC only control group (*n* = 33). Immediately following cast immobilization, both groups participate in four 1-h clinic-based sessions, and a home program for 10 min three times daily until cast removal. Blinded assessments occur within 1 week of cast immobilization (baseline), at three weeks post cast immbolization, cast removal, and at three months post cast removal. The primary outcomes are patient reported wrist/hand function and symptomology on the Patient Rated Wristand Hand Evaluation, McGill Pain Questionnaire, and Budapest CRPS Criteria. The secondary outcomes are grip strength, active range of motion as per goniometry, circumferential edema measurements, and joint position sense.

**Discussion:**

This study will investigate the early effects of mGMI + SOC hand therapy compared to SOC alone. We intend to investigate whether an intervention, specifically mGMI, used to treat preexisiting pain and motor dysfunction might also be used to mitigate these problems prior to their onset. If positive effects are observed, mGMI + SOC may be considered for incorporation into early rehabilitation program.

**Trial registration:**

This trial is registered at ClinicalTrials.gov with identifier NCT02957240 (Approval date: April 20, 2017).

## Background

### Epidemiology, etiology, and medical treatment of DRF

Distal radius fractures (DRF) are the most common fractures of the upper extremity in the United States [1–3]. These fractures account for up to 18% of all fractures in persons over the age of 65 years and are nearly 5 times more likely to occur in women than in men [4]. With advancing age, the incidence for women increases rapidly after age 55, almost doubling every 10 years until 90 years of age [5] with the average age of occurrence being 56 years [6]. Women in an urban setting are 30% more likely to sustain a DRF than those in a rural setting [7]. The risk of sustaining a DRF is 20% greater in winter months (*RR* = 1.2), and 45% more likely to occur on days that begin with snow and ice on the ground (incidence rate ratio, 1.45) [8–10]. Given the globe’s aging population, osteoporotic fractures of the wrist, humerus, spine or hip are expected to increase further in the coming years, and the aftercare will be an increasing burden on healthcare resources [11]. Typical medical care for non-operative DRF involves closed reduction in the emergency department or orthopedic office, followed by immobilization in a forearm based cast for four to six weeks, and early active range of motion to non-immobilized joints [12, 13].

### Function and impairment following DRF

Women sustaining a wrist fracture are likely to have a clinically important functional decline in self care, productivity, and leisure performance [14]. Disability levels based on the Patient Rated Wrist and Hand Evaluation (PRWHE) are reported to be high (75%) at the first week after DRF, and modertate (43%) at 8 weeks post injury [15]. Wrist and forearm active range of motion (AROM) and hand grip strength are the most commonly assessed physical impairments in all persons who are status-post DRF and are, on average, reduced by 40 to 50% at eight weeks post injury [16]. A case-control designed study revealed that distal radius fractures in both men and women have large effects (ƞ^*2*^ = .49) on grip strength relative to matched controls [17] and, in turn, grip strength is a significant predictor of wrist function as measured by the PRWHE [18, 19]. Recent research also suggests that persons with DRF experience significant impairments in sensorimotor functions such as wrist joint proprioception and moving light touch awareness [17]. There are also indications that sensorimotor functions have significant associations (fine motor: *r* = −.52, joint position sense error: *r* = .63) with pain levels [17].

### Type I complex regional pain syndrome and DRF

Resting pain and pain with activity are common aftereffect following DRF and have been documented to persist in 32.6 and 41.9% of cases respectively at two years post injury [20]. At the time of initial follow up after first definitive medical/surgical treatment of DRF, 81% of persons have been reported to describe having “severe” to “very severe” pain [15].

Type I complex regional pain syndrome (CRPS) is a condition that presents following an injury or illness where nerves are not damaged and is characterized by regional pain, increased sensitivity to touch, swelling, and impairment of motor function that are out out of proportion relative to the extent and location of the original injury/illness [21–24], and has been reported to occur in as many as 37% of persons who sustain DRF [25]. Jellad et al. [25] report that women are 5.8 times more likely than men to develop CRPS after DRF. Persons who sustain low to medium energy impact DRF are 7.7× more likely to develop CRPS than those who sustain high impact fractures, [25]. Additionally, Moseley et al. [26] report that persons with a 0–10 numerical pain scale (NPS) rating of greater than 4 within one week after closed reduction and casting for DRF were 15.1 times more likely to develop CRPS, than those with less than a 5/10 NPS rating.

Many theories exist on the mechanisms for developing Type I CRPS [27]. Recent evidence indicates that CRPS likely has a component mediated by the cerebral cortex [28, 29]. Neuroimaging studies have revealed CRPS-associated shrinkage of the areas of the primary somatosensory cortex (S1 and S2) that represent the painful limb [30, 31]. These cortical changes may be linked with pain sensitization [28, 30]. Other researchers have illustrated disinhibition of the motor cortex and a disrupted body schema in persons with CRPS [32, 33]. Peripheral sensorimotor changes such as those observed by Karagiannopoulos et al. [17] may be indicative of cortical changes occurring following DRF and cast immobilization.

As many as 37–58% of persons undergoing closed treatment and cast immobilization following DRF go on to develop Type I CRPS [34, 35]. These numbers justify attention, given that most DRF are managed non-surgically via closed reduction and cast immobilization [36, 37]. Therefore, it is important to tailor therapeutic interventions for DRF that address or prevent development of CRPS. Recent evidence suggests that CRPS may develop due to central nervous system changes [27]. Thus, modulation cortical activity might be a noteworthy therapeutic avenue for individuals at risk of developing a CRPS.

### Movement representation techniques

Interventions designed to address centrally mediated pain and motor dysfunction are referred to as *Movement Representation Techniques (MRT)* [38]. These therapies involve observing or imagining normal and pain free movements, which may be performed simultaneously with sensory stimulation and active motion. The aim of MRT is to facilitate pain free movements of a painful limb [38]. MRT include mirror therapy (MT) and graded motor imagery (GMI). GMI is a multidimensional MRT and consists of three intervention phases: 1) limb laterality recognition, 2) Explicit motor imagery, and 3) the aforementioned, MT. Phase 1 involves viewing images of upper limbs and identifying if they are right or left limbs. In the absence of worsening limb symptomology and after proficiency in identifying limb segments has been established, participants transition into phase 2. Phase 2 involves viewing of images of upper limbs and imagining that the affected limb segment is assuming the postures depicted absent pain. Providing that symptoms are controlled, after 2 weeks participants transition into phase 3. In phase 3 (MT), the mirror image of the unaffected limb is observed engaging in various postures. This phase is completed in 2 weeks. All phases progress participants through imagining, and viewing movements that are least to most likely to proke painful experiences.

#### MRT in CRPS

Current evidence provides support for MRT for pain reduction when used in rehabilitation. A meta-analysis found that MRT were effective in reducing pain (*SMD* = 0.82 and disability (*SMD* = 0.72) in persons with chronic pain syndromes [38]. The authors recommended that MRT, particularly MTand GMI, should be considered for patients with CRPS. The aforementioned MRT, MT, has been successful for persons with acute Type I CRPS [39]. It is suggested that patients activate cortical networks by imagining pain free movement and sensation of the affected limb prior to introducing MT [40, 41]. Persons with chronic type I CRPS who performed GMI experienced decreased pain and swelling [F (1,11) = 57] as early as two weeks following implementation and had sustained positive effects on pain (F(3,46) = 8.701) and function (F(3,46) = 7.327) at 6 months follow-up [40].

#### MRT and DRF

Preliminary evidence supports MRT to address pain and physical function in persons with distal radius fracture. Frenkel et al. [42] evaluated the effect of “explicit mental imagery” on wrist and forearm active range of motion when provided to healthy adults during a period of casted wrist immobilization. Compared to a no-treatment control group, those in the imagery group had significantly better wrist extension [*F* (1,16) = 33.375] and ulnar deviation [*F*(1,16) = 7.776] than controls [41]. A study of patients with finger injuries who performed MRT in addition to traditional occupational therapy (OT) interventions indicated improved ROM as measured by a goniometer (*t* = 7.8) and a reduction in disability as measured by the Disabilities of the Arm, Shoulder and Hand (DASH) questionnaire (*t* = − 4.79) [43, 44].

Bayon-Calatayud et al. [45] carried out a randomized controlled trial in 22 patients with closed DRF managed surgically or conservatively. Two groups completed a conventional OT regime while one (experimental) group also completed MT. The outcomes measured were pain, active wrist extension, and disability. No statistically significant differences were found between groups on these measures. However, the findings were limited by 1) a heterogeneous sample of surgical and non-surgical patients without any mention of the baseline characteristics of either group, 2) participants not being blinded to group assignment, 3) a non-descript treatment protocol, 4) no reporting of the temporality of the initiation of the study relative to the injury, and 5) the use of a unidimensional MRT (i.e., MT only). At present, there is paucity in the literature on the use of MRT to prospectively address pain, sensorimotor dysfunction, and disability in persons with DRF and what exists is limited in by its methodology and intervention unidimentionality.

### Rationale for the present study

The upper limb rehabilitation literature is rich in restorative approaches (i.e., resolving impairments when already present). However, little is known about strategies to prevent upper limb, impairment, disability and pain. Recent longitudinal studies indicate that as many as 1/3 to nearly 1/2 of all persons who undergo closed treatment of DRF develop type 1 CRPS [24, 25] and 80% of these patients are women [25]. Current evidence indicates that cortical changes in upper-limb sensory andsensorimotor representation occur within days or weeks during immobilization [46–48], and these changes appear to mimic cortical changes in persons with type I CRPS. A multidimentional MRT, Graded motor imagery (i.e., laterality training, motor imagery and mirror therapy), has emerging support in the literature as a restorative treatment strategy for patients with CRPS [38, 39, 41, 42, 44], however, studies have not examined GMI as a treatment strategy to prevent the onset of CRPS. Implementation of the present protocol will allow investigation into the effects of a modified GMI (mGMI) intervention during the cast immobilization period for women with closed DRF.

## Methods

### Objectives and hypotheses

#### Primary objective

To determine if women at risk for CRPS development after closed treatment of DRF who participate in a mGMI and SOC hand therapy have differing function, pain, upper limb impairments, and counts of CRPS diagnoses following cast removal, and at 1 and 3 months when compared to those who receive only the SOC.

#### Hypothesis

We hypothesize that the combined GMI/SOC intervention will have a positive effect on disability, pain and upper limb impairments at 1 month following cast removal, and counts of CRPS diagnoses at 3 months post cast removal when compared to SOC alone.

### Design

This is a protocol for a randomized comparative effectiveness trial, where the therapy outcomes of two six-week long intervention programs, aSOC only programand an mGMI+SOC program, are compared. The experimental design will be as is described in Table 1.

**Table 1:** Experimental design

	Baseline	Weeks 1–3	Week 3	Week 4–6	Cast Removal	4 weeks after cast removal	12 weeks after cast removal
R	O_1_	X	O_2_	X	O_3_	O_4_	O_5_
R	O_1_	X	O_2_	X	O_3_	O_4_	O_5_

Key: X = treatment; O_1_ = pretest; O_2_, O_3_, O_4_, O_5_ = posttests; R = randomization

### Participants

**Fig. 1:**
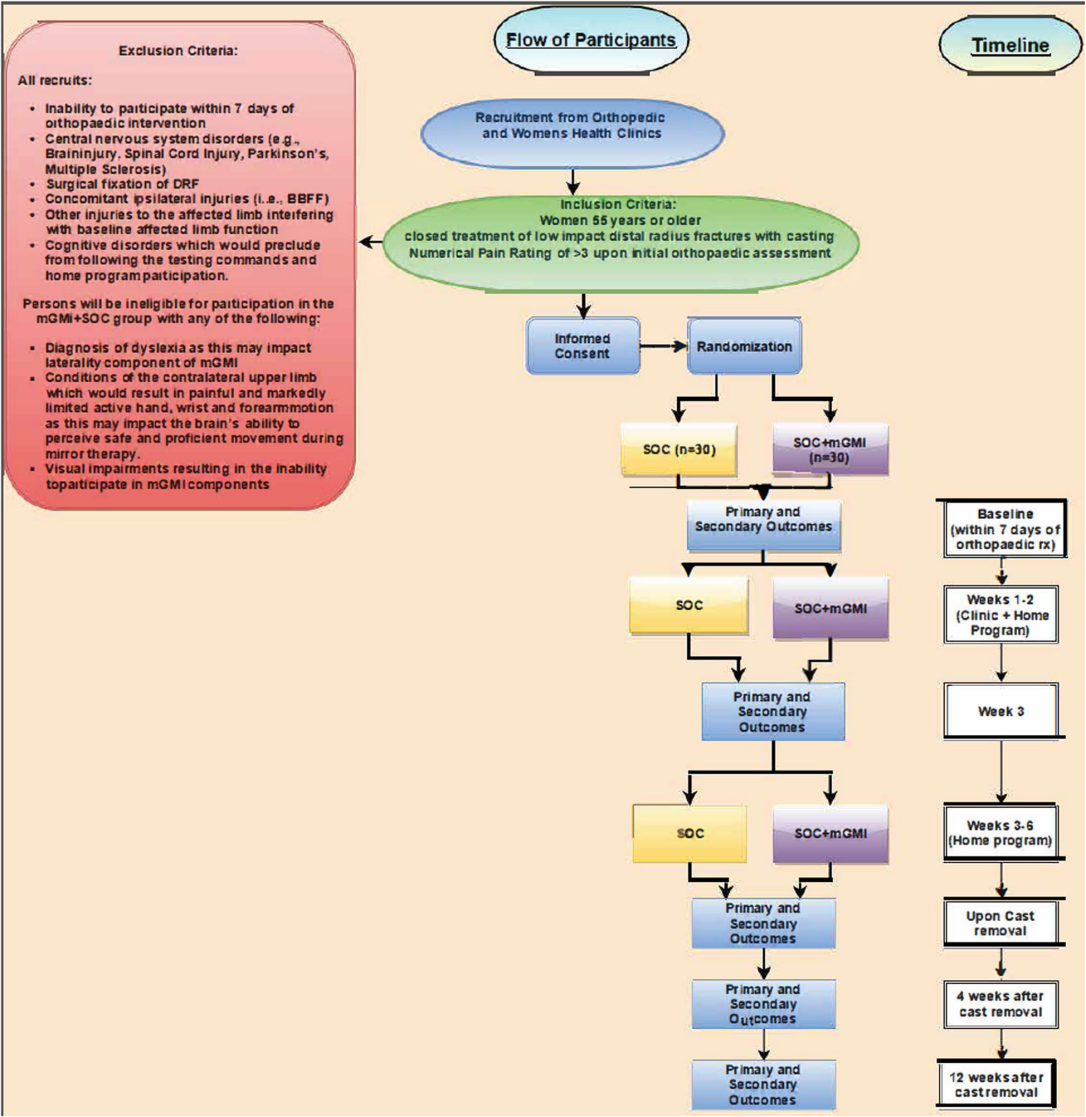
Flow of participants

To ensure that criteria for participation are met, patients will be recruited from outpatient clinics staffed by physicians specializing in Orthopaedic, Sports and Family Medicine. The clinics are associated with a large public university in the Midwestern United States. At the time of the first orthopaedic treatment for closed DRF, patients will be offered the opportunity to participate. Prior to enrollment and allocation, oral and written informed consent will be sought. We will seek to enroll 66 participants who have received closed treatment of a distal radius fracture. Through use of a block randomization method, participants will be allocated to a SOC intervention (*n* = 33) group or a SOC + mGMI (*n* = 33) group. Following the consent process, the PI, not a blinded evaluator or interventionist, will actuate the randomization through use of a random block assignment generator [49]. To ensure partcipants are and remain blinded to allotment, participants will be informed that they will be randomized to one of two “occupational therapy treatment approaches” and will be asked not to discuss their treatments with others enrolled in the trial. Evaluators will be blinded to allocation. The feasibility of enrolling these numbers is high given that from 2013 to 2015, the aforementioned Orthopaedics clinic alone cared for an average of 218 women a year who met the below described inclusion criteria. These numbers are likely attributable to the characteristics of the area served by this health system (i.e., urban, northern latitude with slippery weather conditions) which are associated with increased incidence of DRF [7, 9, 10]. The proposed flow of participants from enrollment to completion is reflected in Fig. 1.

#### Inclusion criteria

The intent of this study is to target persons with high risk for developing CRPS. Therefore, it is limited to women with fragility or low-impact type fractures who are:

- 55 years or older
- managed with closed orthopaedic treatment and cast immobilization
- reporting a numerical pain rating of greater than 3/10 within 1 week of initial medical management of their fracture

These patient factors are known to have strong associations with type I CRPS development [4, 26, 34].

#### Exclusion criteria

The following factors will indicate exclusion from the present study:

- Concurrent rehabilitation services elsewhere (chiropractic, acupuncture, occupational or physical therapy) that address sequelae of forearm fracture
- Surgical fixation of DRF
- Central nervous system disorders (e.g., Brain injury, Spinal Cord Injury, Parkinson’s Disease, Multiple Sclerosis)
- Concomitant ipsilateral injuries such as both bone forearm fractures
- Other injuries to the affected limb interfering with baseline affected limb function
- Cognitive disorders which would preclude the participant from following the testing commands and home program participation
- Diagnosis of dyslexia as this may impact the laterality component of mGMI
- Conditions of the contralateral upper limb which would result in painful and markedly limited active hand, wrist and forearm motion as this may impact the brain’s ability to perceive safe and proficient movement during mirror therapy
- Visual impairments resulting in the inability to participate in mGMI components

### Intervention procedures

DRF will be managed as per the American Academy of Orthopedic Surgeons guidelines [13]. Upon enrollment in the study, participants will be randomized to an mGMI +SOC group or a SOC only group. The SOC group includes a combination of clinic and home based programming to address hand, elbow and shoulder motion as well as edema control whereas the mGMI +SOC group receives the same SOC programming in addition to MRT interventions intended to maintain the affected limb’s cortical representation. Participants in both groups will complete four clinic-based intervention sessions of an hour each across a 6 week period where thefocus will be on facilitating home program competency and advancement as indicated. The first session will occur within one week of cast treatment and home programming will supplement these clinical sessions. The entire therapy protocol for each group is standardized and manualized to ensure intervention fidelity. SOC and all phases of mGMI home programming are standardized and are to be provided in the form of written materials. The format will be one on one with the interventionist and will involve instruction in home programming as described below. All interventions will be carried out by certified OTs, including a certified hand therapist. Table 1 illustrates the intervention timeline. To enhance intervention fidelity both interventions are protocolized and manualized and the evaluator all interventionists will undergo formal training by the PI. Prior to the initiation of the trial, the PI will conduct a competency assessement of the evaluator and the interventionists.

#### Home program (mGMI + SOC or SOC)

SOC consists of the aforementioned 4 one-on-one treatment sessions and a home exercise program [50, 51]. Participants will be instructed to complete their home program three times daily for 15 min. This will consist of AROM for 10 repetitions each of thumb opposition, thumb radial abduction and extension, finger metacarpophalangeal joint flexion with interphalangeal joint extension, tendon gliding series, finger abduction and adduction, forearm supination and pronation, elbow flexion in supination alternating with elbow extension in pronation, “shoulder rolls” for scapular active motion, shoulder flexion to 90 degrees, shoulder internal rotation behind the back with a light dowel, shoulder external rotation with the elbow at 90 degrees and forearm sliding on a table, shoulder abduction with the forearm sliding on a table, and supine shoulder flexion with the dowel. Exercises should be completed within a pain-free arc of movement. The programs’ intensity and duration will conform to the American Academy of Sports Medicine’s guidelines for older adults [52] however will be tailored according to each participant’s unique habits and routines so as to enhance adherence.

Edema will be addressed through elevation above heart when at rest and for 10 min on the hour. When resting in a seated or supine position, participants will be instructed to use pillows to accomplish elevation and to keep elbows in an extended position. Compression will not be recommended due to the linkage between tight casting and CRPS development [18]. Instructions for elevation will be provided immediately following orthopedic intervention. Strict elevation will be recommended for the first 72 h after the injury.

#### Modified graded motor imagery (mGMI) home program

Although the GMI intervention reported by Mosely had significant functional, pain-reducing, and edema-reducing benefits, it may have limited feasibility as it requires performing a home program for 10 min each waking hour. For this reason, Lagueux et al. [53] designed and implemented a modified approach to GMI with three 10 min sessions performed daily. In a six-week program, persons with acute type 1 CRPS reported significantly less pain (*p* = 0.046) on McGill Pain Questionnaire, and improved maximum grip strength measured by dynamometry (*p* = 0.040). The present protocol is based on the modified GMI (mGMI) approach by Lagueax et al. [53] which involved four stages of: 1) Laterality, 2) Explicit motor imagery, 3) Mirror therapy with unaffected hand only and 4) Mirror therapy with bilateral hands. The present study will only involve use the first three phases, given the constraint of cast immobilization. Each phase will be one to two weeks in duration. Participants will be instructed to perform all phases of the mGMI home programming for 10 min, three times a day, six days a week, in addition to performing AROM and elevation as per the SOC. The following describes the mGMI home program of the study:

##### Phase 1 (left/right discrimination)

**Fig. 2:**
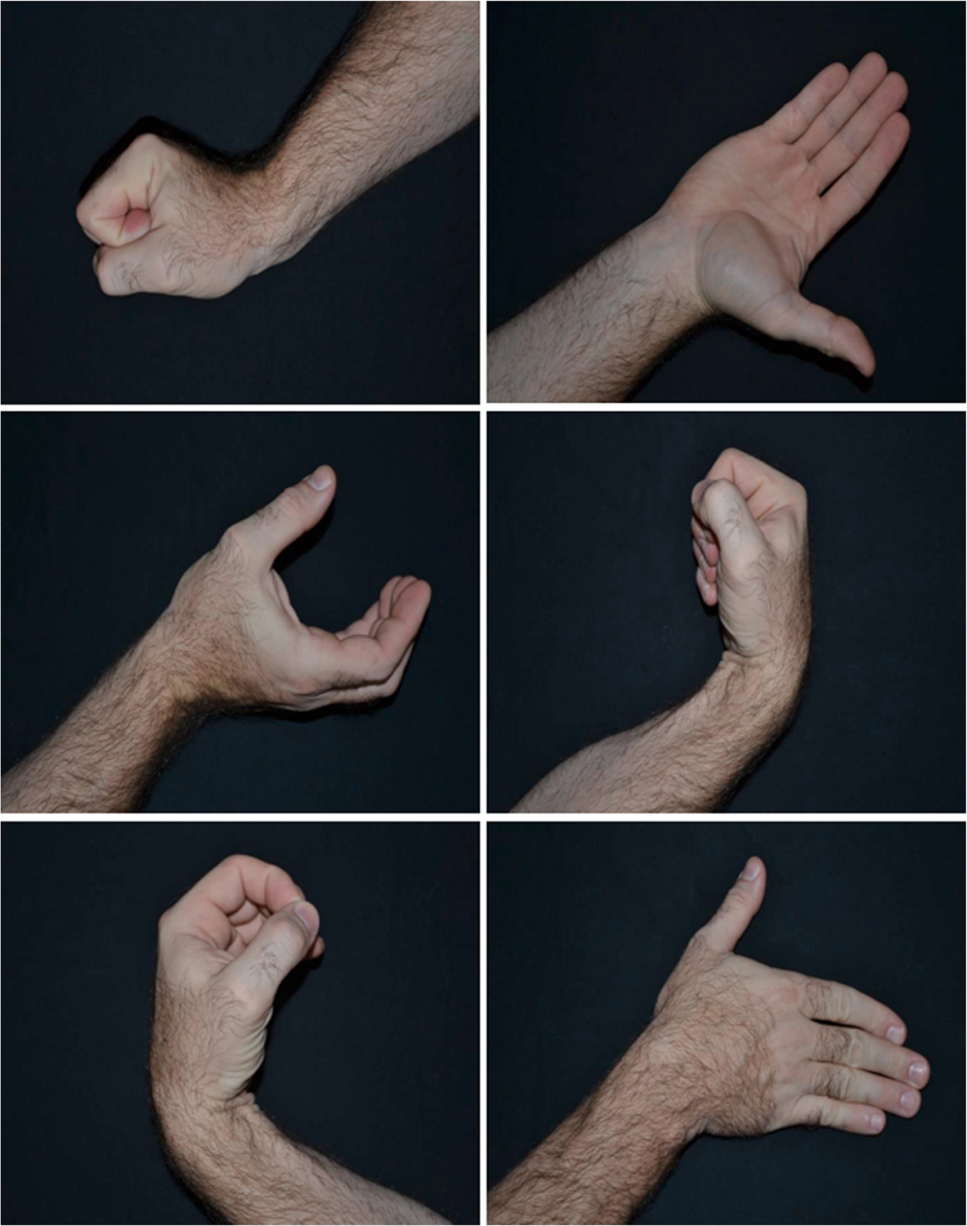
Examples of photos for mGMI phases 1 and 2

When presented with a stack of 50 photos of hands, wrists, and forearms in various postures and orientations (Fig. 2), the participant, without adjusting the cards’ positioning, will determine spontaneously whether it is a right or left upper limb and sort the cards into 2 separate piles. During the clinic session of week two, or when the participant is ready, they will be progressed to the next phase.

##### Phase 2 (explicit motor imagery)

Twenty-five of the 50 images (those which represent the affected side) will be used. In this phase, the participant will select and view a single card and then imagine moving the hand of the casted arm into such a posture and imagine experiencing any relevant sensations including the feeling of movement. The participant will repeat the procedure with all 25 photos at least three times a session. If necessary, to meet the 10 min’ duration, the participant will view magazine photos of an individual performing an activity of meaning. Photos will be chosen that depict the distal aspect of their affected extremity. Upon returning in week four participants will progress to the next mGMI protocol phase.

##### Phase 3 (mirror therapy with mobilization of the non-affected hand)

**Fig. 3:**
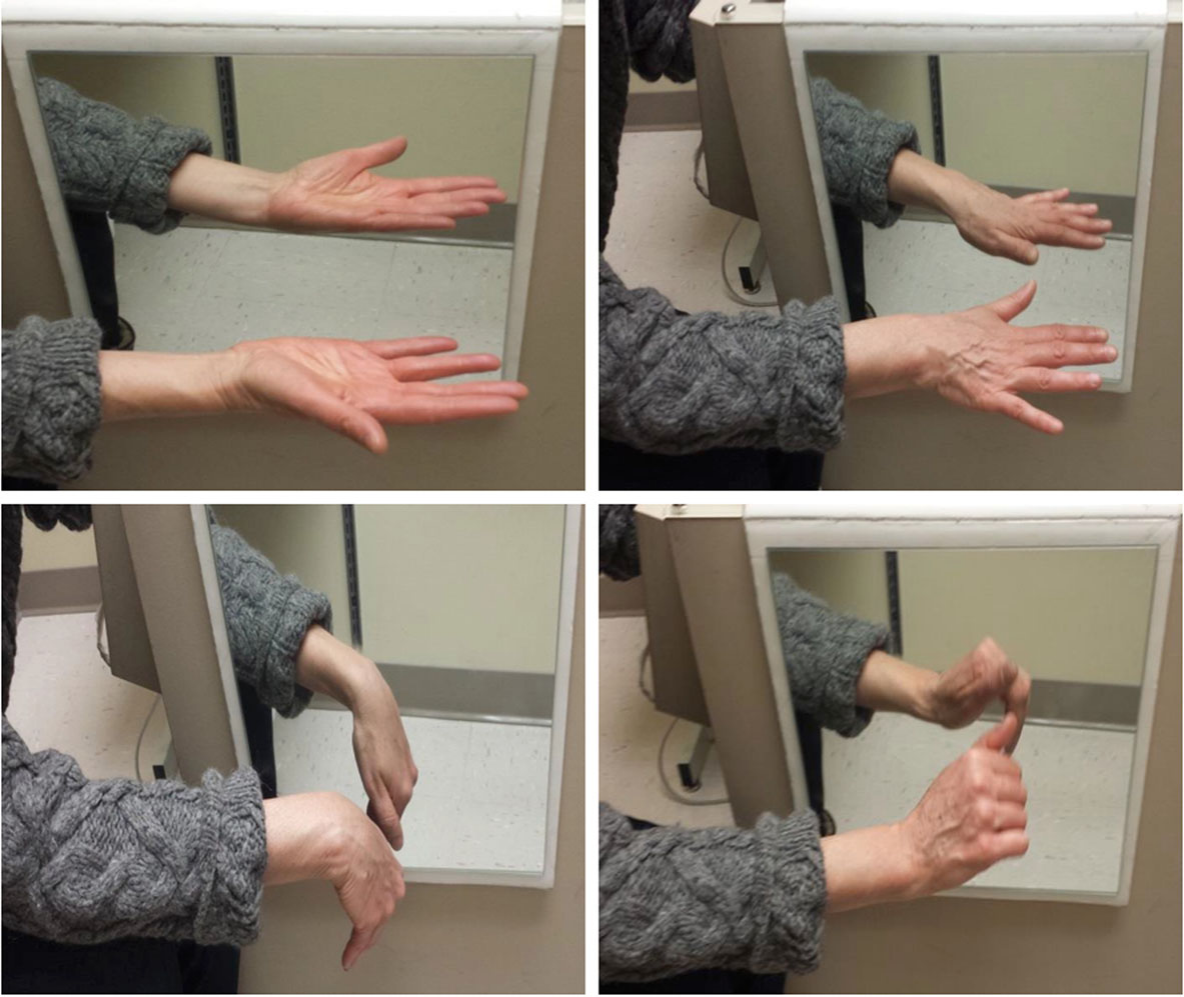
Phase 3: Mirror Therapy

During this phase, the participant will execute the movement depicted by the 25 cards representing the non-affected wrist. They will move the non-affected limb slowly and gently, five to 10 times, while watching the reflection in the mirror. The affected limb remains immobile, hidden in the mirror box. Therefore, the participant views the illusion of moving their injured hand and wrist (Fig. 3*).*

If the participant experiences an increase of more than 2/10 in pain intensity as per the NRS during the mGMI protocol, they will stop and document how long they could participate in that home program session before the increase in pain. There are two options for subsequent sessions: (a) exclude the image that caused the pain or (b) perform the exercise for recorded time minus one minute. The participant will progress to the next phase when exercises do not cause an increase in pain. The interventionist will consider each patient’s pain status prior to progressing her into each new phase. The mGMI protocol, as described above, is that of Lagueux et al. [53] and has been validated for use in persons with Type I CRPS.

### Outcome measurements

All outcome measures will be administered by a single licensed OT who will be blinded to allocation. Blinded assessments occur within 1 week of cast immobilization (baseline), at four three weeks post cast immbolization, cast removal, and at three months post cast removal. A list of measures and a timeline of all data collection points can be found in Table 2.

**Table 2:** Data collection time points

		Baseline (Immediately prior to Starting Therapy)	3 weeks post casting	Week of Cast Removal	4 weeks after cast removal	12 weeks after cast removal
Primary Outcomes	McGill Pain Scale	X	X	X	X	X
	Patient Rated Wrist Evaluation			X	X	X
	Assessment of CRPS I (Budapest criteria)			X	X	X
Secondary Outcomes	Finger Edema (%Unaffected)	X (Affected and Unaffected)	X	X	X	X
	Wrist/Forearm Goniometry (%Unaffected)	X (Unaffected)		X	X	X
	Grip Strength (%Unaffected)	X (Unaffected)			X	X
	Therapy Adherence Log		X	X		
	Patient’s Global Impression of Change			X		
	Continued Hand Therapy Sessions				X	X
	Pain Medication Use	X	X	X	X	X
	Blinding Questionare			X		

#### Primary outcomes

##### PRWHE

The PRHWE [15] is a 15-item questionnaire designed to measure wrist pain and disability in activities of daily living. The PRHWE allows patients to rate their levels of wrist pain and disability from 0 to 10, and consists of 2 subscales: 1) Pain subscale: contains 5 items each of which is further rated from 1 to 10. The maximum score in this section is 50 and minimum 0; 2) Function subscale: contains total 10 items which are further divided into 2 sections i.e. specific activities (having 6 items) and usual activities (having 4 items). The maximum score in this section is 50 and minimum 0. The PRWHE is a widely-used instrument with sound psychometric properties and an established minimum clinical important difference (MCID) after DRF [15, 54, 55] of 11.5 points. The PRWHE has demonstrated higher responsiveness to change after DRF than the DASH questionnaire, as indicated by a higher standardized response mean (SRM) over three and six month periods [55].

##### McGill pain scale

The McGill Pain Scale – Short Form (SF-MPQ), a gold-standard assessment of pain-related affect and intensity [56] will be administered to assess the qualitative and quantitative aspects of pain. The pain rating index has 2 subscales: 1) Sensory subscale with 11 words, and 2) Affective subscale with 4 words from the original MPQ. These items are rated on an intensity scale as 0 = none, 1 = mild, 2 = moderate and 3 = severe. There’s also one item for present pain intensity and one item for a 10 cm visual analogue scale (VAS) for average pain.

Follow up at the mid-point of the intervention (three weeks) is warranted as significant differences in pain have been reported as early as two weeks following the initiation of GMI in persons with CRPS. Additionally, assessing pain at four and 12 weeks post cast removal is justified as this is the time range of onset of CRPS after closed treatment of DRF [26]. Unlike the pain subscales for the PRWHE, the SF-MPQ is not activity-specific, which is of necessity when evaluating the pain of an immobilized upper limb.

##### Budapest CRPS type I diagnostic criteria

A clinical assessment of CRPS presence will be conducted via use of the Budapest CRPS Type I Diagnostic Criteria [57]. These diagnostic criteria were selected over all others due to relatively higher inter-rater reliablity specificity [57]. Given that the timeframe for onset of CRPS after closed treatment of DRF has been found to be one to three months following cast removal [25], participants will be assessed upon cast removal, four weeks post removal and 12 weeks post removal. This assessment will be performed by a board certified orthopaedist with Hand Surgery training. These diagnostic criteria are presented in Table 3.

**Table 3:** Budapest diagnostic criteria

The patient must: 1. Have continuing pain, which is disproportionate to any inciting event 2. Report at least one symptom in three of the four following categories: • Sensory: reports of hyperesthesia and/or allodynia • Vasomotor: reports of temperature asymmetry and/or skin color changes and/or skin color asymmetry • Sudomotor/edema: reports of edema and/or sweating changes and/or sweatingasymmetry • Motor/trophic: reports of decreased range of motion and/or motor dysfunction (weakness, tremor, dystonia) and/or trophic changes (hair, nail, skin) 3. Display at least one sign at time of evaluation in two or more of the following categories: • Sensory: evidence of hyperalgesia (to pinprick) and/or allodynia (to light touch and/or deep somatic pressure and/or joint movement) • Vasomotor: evidence of temperature asymmetry and/or skin color changes and/or asymmetry • Sudomotor/edema: evidence of edema and/or sweating changes and/or sweating asymmetry • Motor/trophic: evidence of decreased range of motion and/or motor dysfunction (weakness, tremor, dystonia) and/or trophic changes (hair, nail, skin) 4. Not have another diagnosis that better explains the signs and symptoms	

#### Secondary outcomes

##### Grip strength

To characterize the generalized hand strength of the sample, maximal voluntary contraction (MVC) strength of the dominant and non-dominant hands will be assessed using a Jamar™ hydraulic hand dynamometer [58]. The Jamar™ has high accuracy, good test-retest reliability [59] and was the measurement device used to collect the adult normative grip and pinch strength data by Mathiowetz et al. [60]. Three trials will be administered per hand via the positioning and verbiage recommended by the American Society of Hand Therapists [61].

##### Wrist and forearm AROM

Active wrist flexion/extension, radial/ulnar deviation and forearm pronation and supination will be measured with a goniometer (AliMed ®, Dedham, MA). The goniometer’s intra-rater reliability when taking measures of active range of motion has been established for of forearm pronation (*ICC* = .83–.86) and supination (*ICC* = .90–.93) [62] and wrist flexion (*ICC* = .96) and extension (*ICC* = .96) [63].

##### Finger edema

Circumferential measurement of the index and long finger first phalanx will be assessed as described by King [64]. In this technique, a calibrated force gauge is affixed to a Jamar™ Finger Circumference Gauge [65] and a circumferential measurement is taken when the tape is pulled with 500 g of force. This approach results in significantly less error between ratings [*F* (1,91) =15.63, *p* < 0.01] than traditional circumferential measurements, likely due to the standardized tension [64]. In general, test re-test reliability is excellent for circumferential measurements of the distal hand (*ICC* = .91) [66]. The index and long digits were chosen because Moseley used this as an outcome of a GMI intervention study and reported significant changes in digital edema. The gold standard of volumetric displacement will not be possible due to cast immobilization [67].

##### Wrist joint position sense

Wrist Joint Position Sense (JPS) testing will be used to measure conscious proprioception, a component of the sensorimotor (SM) system [68]. Karagiannopoulos et al. [69] performed wrist JPS testing in patients following closed and operative DRF and found high responsiveness in the ability of their procedure to detect change in SM function at 8 and 12 weeks following the medical intervention for DRF. The present study will use the same JPS testing protocol. The participant’s JPS is tested in two trials, with their elbow resting on a table, and eyes closed [69]. With vision occluded, the wrist is passively placed in a reference angle of 20° of extension, held for three seconds, and the participant is asked to reproduce the angle. Their final position is measured using standard goniometry procedures, and absolute values are calculated in relation to the reference angle. The statistically significant minimum detectable change (MDC) has been found to be 4.28° to 4.94° [69].

##### Retention, therapy adherence, medication use, and necessity of continued therapy

Notes will be kept regarding the number of women who are screened for the study, as well as the number who are eligible and consent to participation. Data regarding retention (follow-up rates), participant session attendance, adherence to home programming, pain medication use, and costs will be recorded. The retention goal is to have 100% of participants evaluated for the primary outcomes; an evaluation rate of 90% will be considered acceptable. A home therapy adherence log adapted from Brewer et al. [70] and a pain (prescribed and nonpresecribed) mediation use diary adapted from Van Berge Henegouwen et al. [71] will be reported weekly to interventionists. Data on necessity (yes/no) and frequency of continued therapy following cast removal will also be recorded.

##### Protocol fidelity

The PI will oversee regularly scheduled checks of both our outcome assessors and our interventionists. At each check, the PI will use a protocol checklist to document the number of evaluation and intervention protocol deviations and provide real-time feedback.

##### Blinding

To evaluate the effectiveness of the blind, participants and the evaluator will complete a questionnaire related to their opinion of the allocation at the time of cast removal.

### Statistical analysis

Regarding the continuous primary outcomes (i.e., SF-MPQ, PRWHE) a sample size of 30 per group will give 81% power to identify as significant a mean difference of .75 SD in change in these outcomes between the two groups [72, 73]. A sample size of 30 per group will give 81% power to identify as significant a mean difference of 0.75 SD in change in continuous outcomes between the two groups. A sample size of per group will give 81% power to identify 20% vs. 58% as statistically significant for the Dichotomous outcome, Budapest diagnostic criteria. To adjust to an anticipated dropout rate of 10%, we will overenroll in each group by 3 bringing each group’s enrollment to 33. Following this adjustment, the total sample size will be 66 patients.

Data on recruitment, retention, therapy attendance, medication use, continuance of therapy beyond cast removal, and home program adherence will be expressed in terms of rates (e.g., rate of eligibility and randomization for those screened, rates of retention up to and including the final evaluation, and rates of satisfactory adherence to the intervention protocol). Simple descriptive statistics will be employed. Baseline demographics and characteristics will be summarized and compared between groups using Fisher’s exact test for categorical variables and, depending on whether or not parametric assumptions are met, either a two-sample *t*-test or Mann-Whitney U test for continuous variables.

Continuous variable outcomes (Primary: PRWHE, SF-MPQ; Secondary: Goniometry, grip dynamometry and JPS) will be analyzed using a mixed effects linear models to evaluate change over time between groups. Models will include a random effect to account for the within-subject correlation for repeated measurements, and fixed effects of group, time, and group by time interaction. Model assumptions will be examined before fitting models. If assumptions are not met, nonparametric test (Wilcoxon test) will be used. For dichotomous outcomes (diagnosis of CRPS), Fisher’s exact test will be performed to compare proportions between groups.

In a manner consistent with that described of Walenkamp et al. [73], an achor-based approach to determining the MCID of the PRWHE and SF-MPQ will be taken through use of the Patient’s Global Impression of Change (PGIC) [74] scale as the anchor. The PGIC is a seven-point Likert scale, ranging from ‘very much improved’ to ‘very much worse’, and will be administered at the time of cast removal. The Minimal detectable change (MDC) of these two measures will be determined through use of the statistical methods described by Walencamp et al. [73].

All statistical analyses will be conductedby a PhD biostatistician who will employ the use of the Statistical Analysis System software (SAS, version 9.3, 2011; SAS Institute, Cary, NC). All analyses will be performed on an intent-to-treat basis. Two-sided tests with *p*-value less than 0.05 will be considered statistically significant.

## Data and safety monitoring plan

This proposed study involves human subjects. Human subjects are required for this study as we plan to test the repeatability of a measurement tool in adult women. This study cannot be carried out in an animal model nor does it pose any significant risks to the health and wellbeing of human subjects.

Types and magnitudes of risks: 1) Participants might experience some mild hand fatigue or joint aches for 2–3 days following testing and at the onset of the intervention as a result of the repetitive nature of the tasks involved, 2) participants will be asked to give some information that they may be perceived to be of a personal nature and 4) At times, it may be necessary for the researchers to physically touch the participants’ hands or sides to ensure that they are in the correct positions. This, to some, may be uncomfortable.

### Nature and adequacy of protection against risks

Participants will be allowed frequent rest breaks to limit any amount of fatigue and soreness participants may experience in their hands and arms following testing. If discomfort increases as a result of testing, participants will be instructed on self- managing symptoms through use of physical agents such as cryotherapy and rest. In the event that this research activity results in an injury, treatment will be available, including first aid, emergency treatment and follow-up care as needed. Participants who believe they have suffered a research related injury, will be asked to inform the researchers immediately.

If participants are physically uncomfortable or uncomfortable with the nature of the questions or occasional touch, they will be instructed that they are free to withdraw from participation at any time without any impact to relationships with the university.

### Adverse events

At each contact with the participant, the will seek information on adverse events by specific questioning and, as appropriate, by examination. Information on all adverse events will be recorded immediately in the source document, and also in the appropriate adverse event module of the case report form (CRF). All clearly related signs, symptoms, and abnormal diagnostic procedures results will be recorded in the source document. Reports of all serious adverse events (including follow-up information) will be submitted to the IRB within 10 working days if it falls under the UPIRTSO guidelines. Copies of each report and documentation of IRB notification and receipt will be kept in the Clinical Investigator’s binder. A second copy will be sent to the sponsor.

The PI will be responsible for 1) collecting, reporting, and risk management of adverse events, 2) data collection, entry, transmission and analysis, 3) site coordination and enrollment, 4) regulatory issues such as IRB actions, and conflict of interest disclosures, and 5) reporting the interim analysis to IRB. All collaborators will be immediately notified of any adverse events occur.

### Data management plan

REDCap will be used for data capture and management. REDCap is a web-based data entry package that is structured so that access is only through a secure login by certified study personnel. Data entry screens will mimic the format of case report forms and include programmed automatic data field checks for real-time data quality control.

Participant identifiers will be stored separate from raw data in a separate secured and encrytped dataset housed within the RedCAP system. In a separate dataset, participants’ names and date of birth will be linked to a participant number. Demographic data, medical comorbidities data, and outcomes data will be housed in a separate dataset in RedCAP and these data will be linked to these participant numbers.

## Discussion

The long-term goal of the present project and related future work is to help support the health function of women at risk of developing CRPS following DRF through reducing risks associated with CRPS development, sensorimotor dysfunction, and subsequent disability. Should the proposed intervention prove successful in ameliorating the burden of disability, sensorimotor dysnfunction, and CRPS on women after DRF, an ever-growing population of women with DRF will have available to them a non-invasive, and non-pharmaceutical intervention approach which will enhance function, enhance self-management of symptoms, enhance recovery time, be cost-effective, and potentially help to prevent the onset of CRPS.

### Limitations and future study

This protocol is not without some limitations. Participants will not be screened for motor imagery abilities. This was a decision made to avoid use of time-consuming research tools, avoid further restricting elgibility, and because persons with cognitive and right-left discrimination impairments (i.e., Dyslexia) who are subsequently predisposed to challenges with GMI will be screened out. For ethical reasons, the control group in the proposed study will receive standard care and thus, this study does not invole a no-treatment or sham comparison group.

Future study would include additional randomized control trials to investigate 1) the most effective treatment intensity and duration for the protocol, and 2) the combined effectiveness of this and other rehabilitative interventions.

### Project timeline

This study is expected to conclude in late 2021.The initial phase of study preparation has focused on building communication with referral sources to allow for recruitment and ease of enrollment. Pre-trial preparation has focused on interventionist training, refining intervention scripts to deliver patient education consistently, and construction of necessary intervention materials (e.g., portable mirror boxes and laterality cards). Table 4 describes project deadlines.

**Table 4:** Project timeline

	Summer 2017	Fall 2017	Spring 2018-Spring 2021	Summer 2021	Fall 2021
IRB/Clinical Trials.gov registration	x				
Equipment/supplies procurement	x	x			
Interventionist and Evaluator Training		x			
Recruitment/Enrollment			x		
Data Collection			x		
Analyze				x	
Manuscript Submission					x

## Abbreviations

AROM: Active range of motion
CRPS: Complex regional pain syndrome
DASH: Disabilities of the arm, shoulder and hand
DRF: Distal radius fracture
GMI: Graded motor imagery
JPS: Joint position sense
MCID: Minimum clinically important difference
MDC: Minimum detectable change
mGMI: Modified graded motor imagery
MRT: Movement representation techniques
MT: Mirror therapy
MVC: Maximal voluntary contraction
OT: Occupational therapy or occupational therapist
PRWHE: Patient rated wrist and hand evaluation
SF-MPQ: McGill pain questionnaire – short form
SM: Sensorimotor system
SMD: Standardized mean difference
SRM: Standardized response mean
USF: Ulnar styloid fracture
